# Supplementary material for: Specificities of exosome versus small ectosome secretion revealed by live intracellular tracking of CD63 and CD9
Source: Nat Commun. 2021 Jul 19;12:4389. doi: 10.1038/s41467-021-24384-2 (PMC8289845; doi:10.1038/s41467-021-24384-2)
Supplement: Supplementary file 1 — Supplementary Information [file 41467_2021_24384_MOESM1_ESM.pdf]

Specificities of exosome versus small ectosome secretion revealed by live intracellular tracking of CD63 and CD9

Authors
Mathilde Mathieu[1,2], Nathalie Névo[1], Mabel Jouve[3], José Ignacio Valenzuela[4], Mathieu Maurin[1], Frederik Verweij[5], Roberta Palmulli[2,4], Danielle Lankar[1], Florent Dingli[6], Damarys Loew[6], Eric Rubinstein[7], Gaëlle Boncompain[4], Franck Perez[4], Clotilde Théry[1*]

A source data file is provided as a single excel file containing one individual sheet with raw data and/or uncropped Western blot images for each relevant figure panel. This concerns the following figures: Figures 1a-b-c, 2b-c-d, 3c, 4a-b, 5a-b-c-d, 7a-b-c-d-e, 8, supp figures 1a-b, 2a-b, 3a-b-c, 4a-b-c-d-e.

Supplementary Figure 1

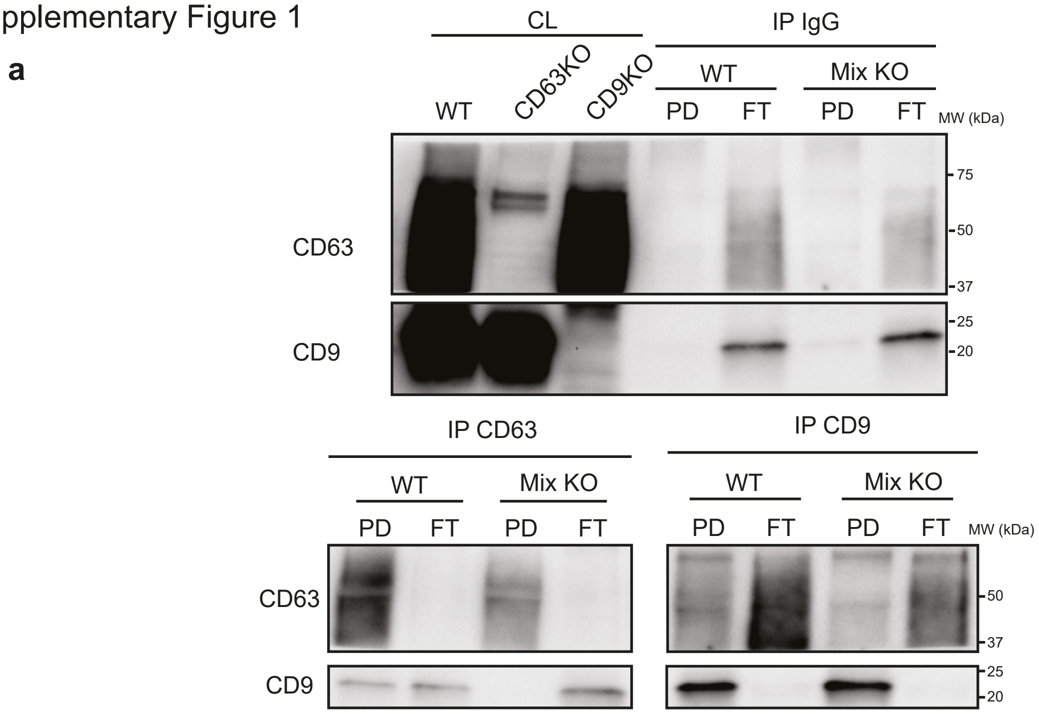

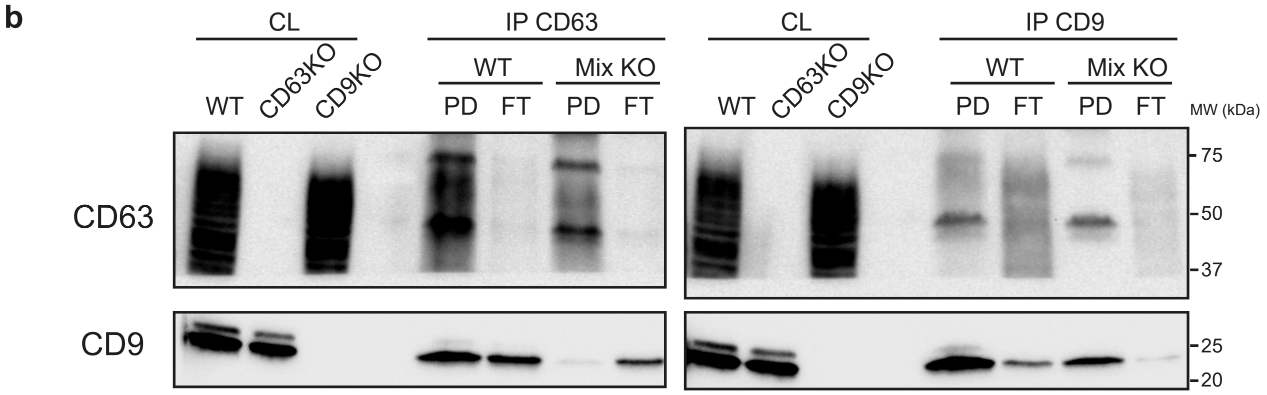

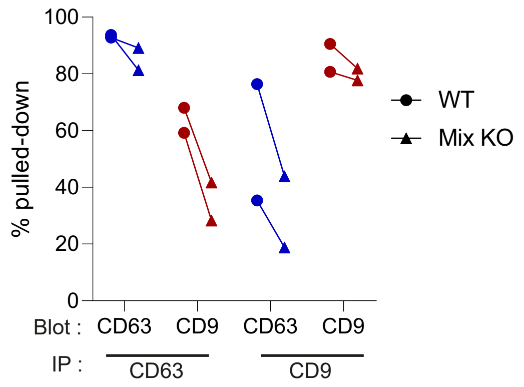

Supp figure 1: control of aggregation of EVs during isolation and immunoprecipitation

a) Western blot of the cell lysates (CL) of HeLa WT, *CD63*-KO and *CD9*-KO, and of the pull-down (PD) and flow-through (FT) after immunoprecipitation (IP) by irrelevant IgG, anti-CD63 or anti-CD9, of HeLa concentrated conditioned medium (CCM) (WT), as compared to a 1/1 mixture of HeLa *CD63*-KO and HeLa *CD9*-KO CCM from the same total number of secreting cells (WT EVs from 20.10^6 cells, mixture of EVs from 10.10^6 *CD63*-KO and 10.10^6 *CD9*-KO cells). Same experimental conditions as in figure 1c (in-house conjugated protein A beads). This experiment was performed once.

b) Similar experiment as in a) performed with anti-CD9 and anti-CD63 beads from Miltenyi (no IgG control beads available), on CCM from HeLa WT or mixed *CD9*-KO and *CD63*-KO. The % of pulled-down CD63 and CD9, calculated based on the bands intensities in the PD and the FT for each condition is represented. Results from 2 independent experiments are shown.

Supplementary Figure 2

a

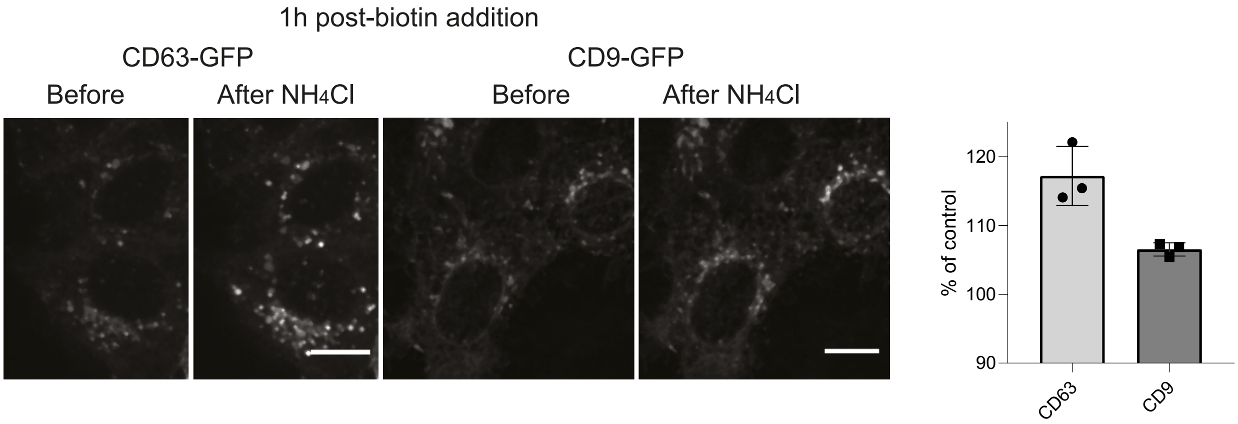

b

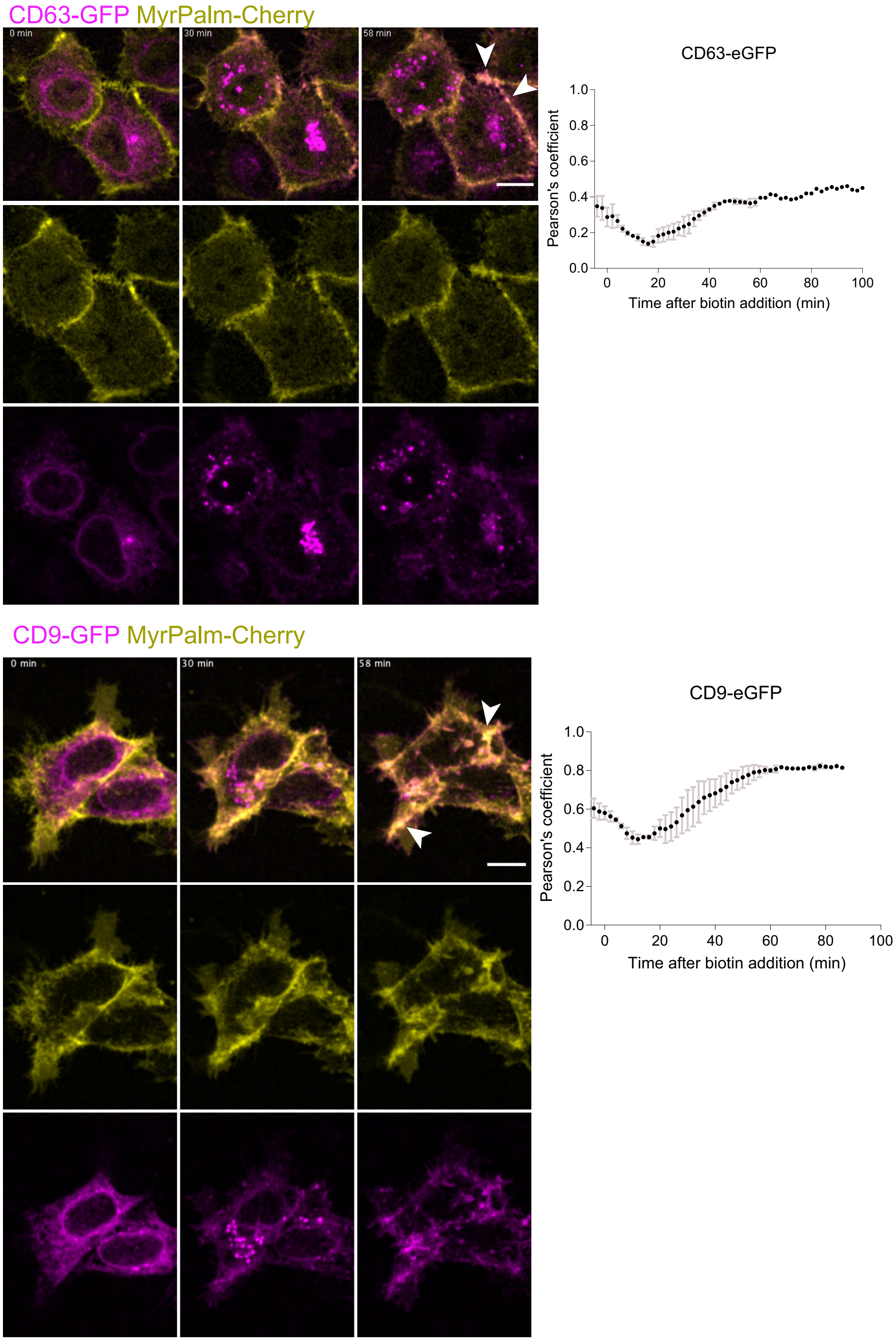

Supp figure 2: Live visualization of acidic compartments and PM by, respectively, NH_4Cl treatment and MyrPalm-mCherry.

a) Micrographs and quantification of live imaging of HeLa cells transfected with the RUSH plasmid *CD63-eGFP* before and after addition in the medium of NH_4Cl at 50mM, 1h after biotin addition. Quantification of the mean fold change +/- SD after NH_4Cl addition of the total GFP fluorescence intensity in 3 independent experiments. 5 fields per experiment were imaged, for a total of at least 10 individual cells to analyze per experiment. Z-projection of 11 planes. Scale bar: 5µm.

b) Micrographs of HeLa cells co-transfected with *CD63*- or *CD9-eGFP* RUSH plasmids and *MyrPalm-mCherry*. Biotin was added at T=0. Z-projection of 11 planes. Scale bar: 5µm. White arrows indicate some sites of co-localization. The median and range of Pearson's co-localization coefficient between eGFP and mCherry is represented over time after biotin addition. The co-localization was quantified in two independent experiments. 5 fields per experiment where imaged, for a total of at least 10 individual cells to analyze per experiment.

Supplementary Figure 3

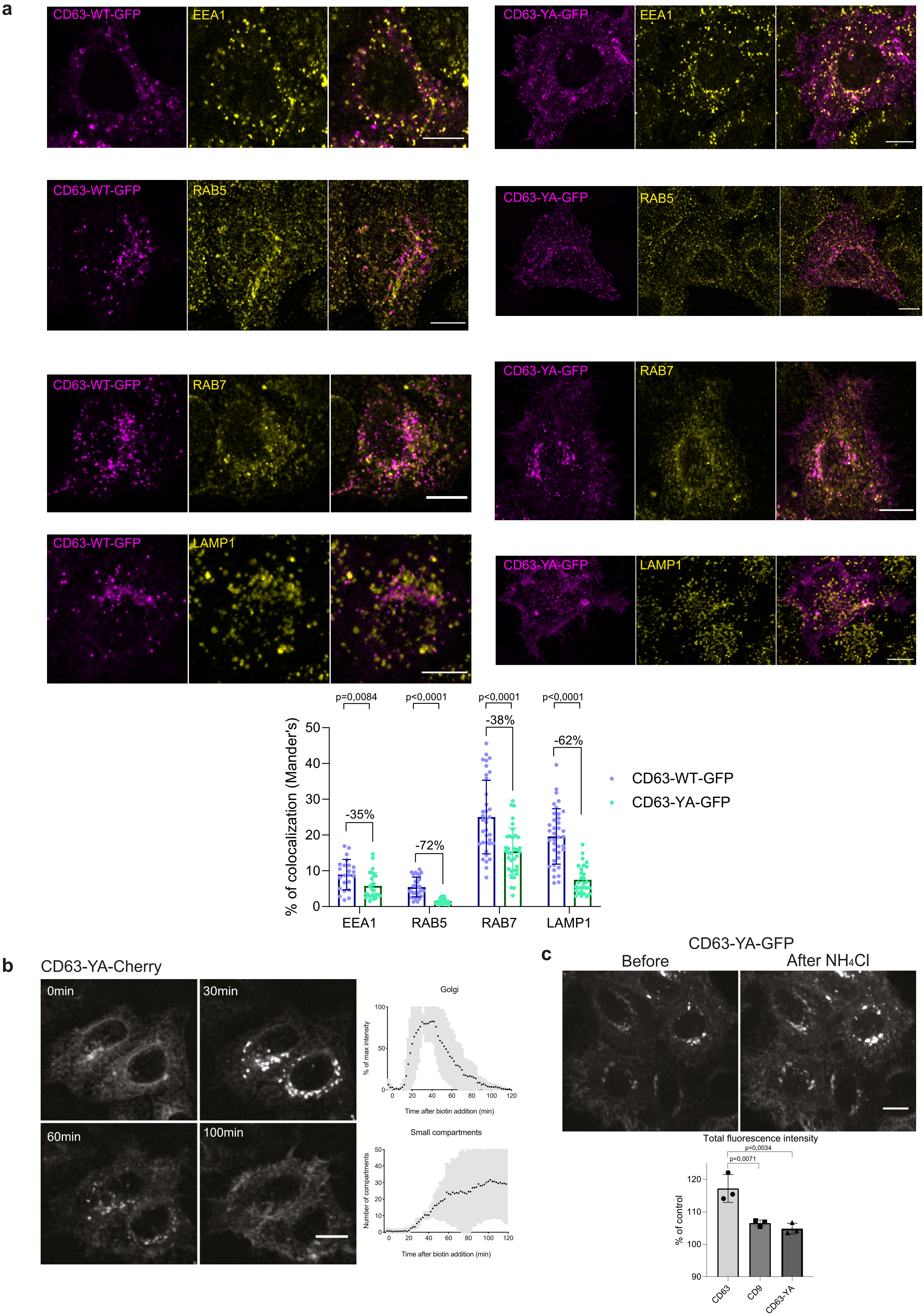

Supp figure 3: localization and trafficking of CD63-YA

a) Representative images (z-projection) and quantification of the co-localization coefficient (Mander's) of RUSH CD63-WT-eGFP and CD63-YA-eGFP with endogenous EEA1, RAB5, RAB7 and LAMP1, stained by immunofluorescence. Mander's coefficient expresses here the percentage of GFP (CD63-WT or CD63-YA) positive for the stained endogenous proteins. Scale bar: 10µm. Pooled results from two independent experiments, each dot represents one cell and the mean +/- SD is represented. Total number of cells used for quantification : EEA1 CD63-WT n=23, EEA1 CD63-YA n=25, Rab5 CD63-WT n=29, Rab5 CD63-YA n=29, Rab7 CD63-WT n=36, Rab7 CD63-YA n=35, Lamp1 CD63-WT n=38, Lamp1 CD63-YA n=29. Two-tailed unpaired t-test.

b) Micrographs and quantification of live imaging of HeLa cells transfected with the RUSH *CD63-YA-mCherry* plasmid. Biotin was added at T=0. Z-projection of 11 planes. Scale bar: 5µm. Quantifications in two independent experiments. 5 fields per experiment where imaged, for a total of at least 10 individual cells to analyze per experiment. The automatically quantified mean Cherry fluorescence intensity in the Golgi +/- SD and mean number of Cherry positive small compartments +/- SD are represented.

c) Extracts and quantification of live imaging of HeLa cells transfected with the RUSH plasmid *CD63-YA-eGFP* before and after addition in the medium of NH_4Cl at 50mM, 1h after biotin addition. Z-projection of 11 planes. Scale bar: 5µm. Quantification of the mean fold change +/- SD after NH_4Cl addition of the fluorescence intensity into GFP positive large compartments in 3 independent experiments. 5 fields per experiment where imaged, for a total of at least 10 individual cells to analyze per experiment. Results from supp figure 2a (CD63-WT, CD9) and CD63-YA are compared. Ordinary One-way ANOVA, Tukey's multiple comparisons test.

Supplementary Figure 4

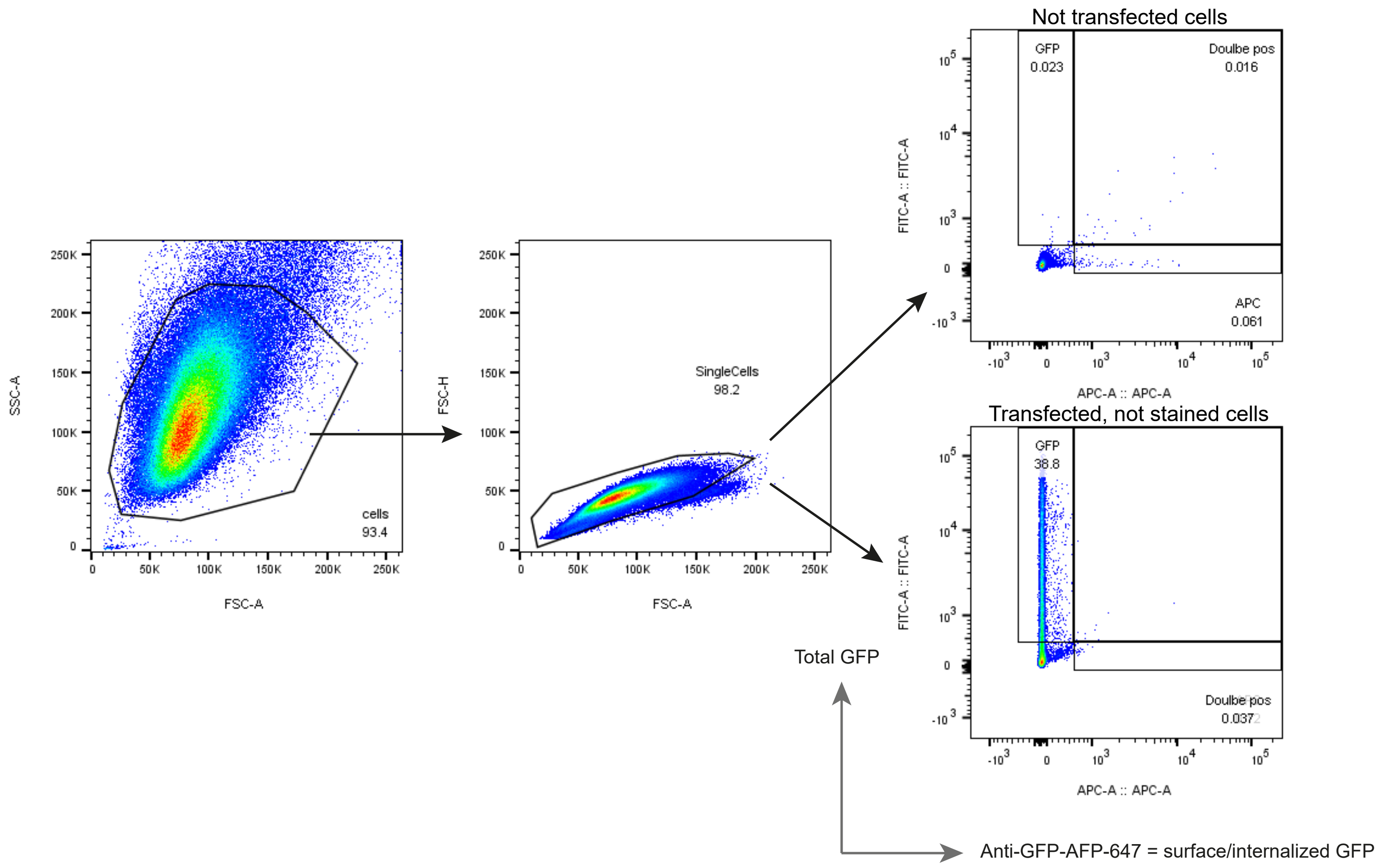

Supp figure 4: gating strategy for flow cytometry based-analysis of surface exposure of RUSH constructs.

Cells expressing GFP-RUSH constructs and secondarily labeled with anti-GFP-AF647 where gated 1) to exclude debris based on FCS/SSC, 2) to exclude doublets based on FSC-A/FSC-H, followed by analysis for GFP and AF647 label, as shown in figures 4a and 4b. The example shown here corresponds to control cells of figure 4a.

Supplementary Figure 5

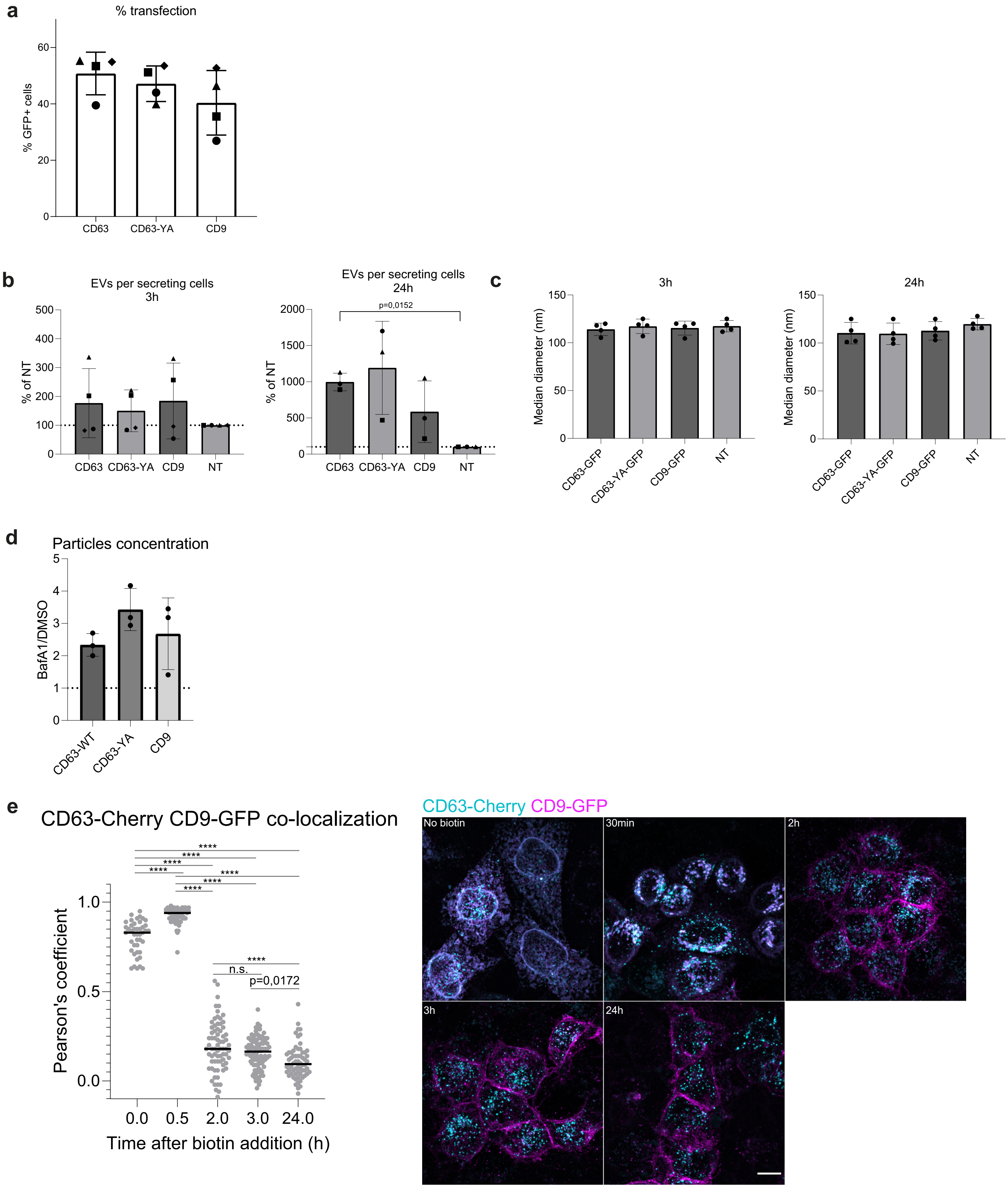

<u>Supp figure 5: controls of transfection efficiency, EVs concentration and size of RUSH-*CD63-eGFP* and -*CD9-eGFP* transfected cells, treated or not with BafA1, and localization of CD63 and CD9 at 3h and 24h of trafficking</u>

a) Percentage of GFP positive cells transfected with the RUSH *CD63-eGFP*, RUSH *CD63-YA-eGFP* or RUSH *CD9-eGFP* constructs, measured by flow cytometry, represented as mean +/- SD of 4 independent experiments. No significant difference observed with an ordinary One-way ANOVA, Tukey's multiple comparisons test.

b) Number of EVs secreted per cell in each condition, at 3h (left) or 24h (right) after biotin addition, quantified by NTA, normalized to the NT sample. Mean +/- SD of n=3 (24h) or 4 (3h) independent experiments. Ordinary One-way ANOVA, Tukey's multiple comparisons test.

c) Median size of EVs secreted per cell in each condition, at 3h (left) or 24h (right) after biotin addition, quantified by NTA in 4 independent experiments, the mean +/- SD is represented. No significant difference observed with an ordinary One-way ANOVA, Tukey's multiple comparisons test.

d) Ratio BafA1/DMSO of the concentration of particles measured by NTA for each sample of figure 5c. Results for 3 independent experiments, and the mean +/- SD are represented. Ordinary One-way ANOVA, Tukey's multiple comparisons test, non-significant difference between the conditions.

e) Representative confocal images and Pearson's colocalization coefficient of RUSH CD63-mCherry and RUSH CD9-eGFP in transfected cells fixed at different time points after biotin addition. One dot represents one cell, the median is represented (black line). Between 5 and 10 fields were imaged to quantify at least 12 cells per condition per experiment, in 3 independent experiments. Ordinary One-way ANOVA, Tukey's multiple comparisons test ****: $p<0,0001$. Scale bar: 10μm.

Supp table 1 : sequences used for generating plasmids.

For *CD9* and *CD81* genes: N-terminal sequences, restriction sites in bold, linker in italics.

For MyrPalm plasmid: MyrPalm sequence in bold.

Synthetic genes	
CD9 N-term	ATGcctCCGGTCAAAGGAGGCACCAAGTGCATCAAATACCTGCTGTTCGGATTTAACTTCATCTTCTGGCTTGCCGGGATTGCTGTCCTTGCCATTGGACTATGGCTCCGATTCGAC**gaattc**ACCGGT**ggccggcc**A*ACCGGTGGAGCTCGAATCAGATCT*TCTCAGA…
CD81 N-term	ATGcctGGAGTGGAGGGCTGCACCAAGTGCATCAAGTACCTGCTCTTCGTCTTCAATTTCGTCTTCTGGCTGGCTGGAGGCGTGATCCTGGGTGTGGCCCTGTGGCTC**gaattc**ACCGGT**ggccggcc**a*ACCGGTGGAGCTCGAATCAGATCT*CGCCATG…
Primers	
CD63-YA forward	TGAAGAGTATCAGAAGTGGC**GC**CGAGGTGATGTAGttaatT
CD63-YA reverse	AattaaCTACATCACCTCG**GC**GCCACTTCTGATACTCTTCA
Other sequence	
MyrPalm and linker	ATG**GGCTGCATCAAGAGCAAGCGCAAG**GACAACCTGAACGACGACGGCGTGGACgaaccggtcgccacc
sgRNA for CRISPR/Cas9 KO	
CD63	CCAGTGGTCATCATCGCAGT
CD9	GAATCGGAGCCATAGTCCAA
