# Supplementary material for: Specificities of exosome versus small ectosome secretion revealed by live intracellular tracking of CD63 and CD9
Source: Nat Commun. 2021 Jul 19;12:4389. doi: 10.1038/s41467-021-24384-2 (PMC8289845; doi:10.1038/s41467-021-24384-2)
Supplement: Supplementary file 2 — Description of Additional Supplementary Files [file 41467_2021_24384_MOESM2_ESM.pdf]

Description of Additional Supplementary Files

File Name: Supplementary Data 1
Description: Results of the quantitative mass spectrometry of the GFP samples compared to the control NT samples. The raw proteomics data are available on ProteomeXchange at PXD021515

File Name: Supplementary Data 2
Description: Results of the quantitative mass spectrometry of the CD63-eGFP samples compared to the CD9-eGFP samples. The raw proteomics data are available on ProteomeXchange at PXD021515.
